# Process optimization and effect of thermal, alkaline, H_2_O_2_ oxidation and combination pretreatment of sewage sludge on solubilization and anaerobic digestion

**DOI:** 10.1186/s12896-020-00614-1

**Published:** 2020-05-06

**Authors:** Salar Siami, Behnoush Aminzadeh, Razieh Karimi, Seyed Mostafa Hallaji

**Affiliations:** 1 School of Environment, College of Engineering, University of Tehran, Tehran, Iran; 2 Gorgan University of Agricultural Sciences & Natural Resources, Golestan, Iran; 3 Faculty of Engineeringss, Department of Civil Engineering, Monash University, Melbourne, Australia

**Keywords:** Anaerobic digestion, Sewage sludge, Pretreatment, Methane production, Response surface methodology

## Abstract

**Background:**

This study investigated the feasibility of enhancing anaerobic digestion of sewage sludge with triple, dual, and individual pretreatment of waste activated sludge with heat, alkalinity, and hydrogen peroxide. These pretreatments disrupt sludge flocs, organisms’ cell walls, extracellular polymeric substance, and intracellular organic matter, which increase biodegradability and hydrolysis rate of activate sludge. In addition, the influence of various variables on methane production was analyzed using the response surface methodology with the quadratic model. Eventually, an optimized temperature and chemical concentration for the highest methane production and lowest chemical usage is suggested.

**Results:**

The highest amount of methane production was obtained from the sludge pretreated with triple pretreatment (heat (90 °C), alkaline (pH = 12), and hydrogen peroxide (30 mg H_2_O_2_/g TS)), which had better performance with 96% higher methane production than that of the control sample with temperature of 25 °C approximately and a pH = 8. Response surface methodology with a quadratic model was also used for analyzing the influence of temperature, pH, and hydrogen peroxide concentration on anaerobic digestion efficiency. It was revealed that the optimized temperature, pH, and hydrogen peroxide concentration for maximizing methane production and solubilization of sludge and minimizing thermal energy and chemical additives of the pretreatments are 83.2 °C, pH = 10.6 and 34.8 mg H_2_O_2_/g TS, respectively, has the desirability of 0.67.

**Conclusion:**

This study reveals that triple pretreatment of waste activated sludge performed better than dual and individual pretreatment, respectively, in all desirable output parameters including increasing methane production as the most important output, increasing in COD solubilization, protein and polysaccharide, and decreasing in VSS solubilization.

## Background

Energy supply is one of the most important challenges in today’s world. Nowadays, the most important source of energy production, especially in developing countries, is fossil fuels due to their easy availability and low cost. However, fossil fuels have many problems, including environmental problems and non-renewability. Therefore, the need for renewable and clean energy is rapidly increasing [1]. On the other hand, contamination caused by human activity has created numerous problems for the environment such as climate change, ozone depletion, plant and aquatic species extinction, and water contamination [2]. One way to address these issues is the use of wastewater treatment plants, in which wastewater is treated prior to being released into water streams. An important challenge associated with wastewater treatment plants is the management of sludge produced from the treatment process. This is important because sludge treatment units are the most cost-intensive parts of wastewater treatment plants, accounting for up to 60% of their total operating costs [3–5].

As a practical solution for simultaneous renewable energy production and contamination removal from the sludge treatment process, anaerobic digestion of sewage sludge has been used widely in wastewater treatment plants [6]. Anaerobic digestion of sewage sludge includes four predominant reactions, namely hydrolysis, acidogenesis, acetogenesis, and methanogenesis. Hydrolysis reaction plays an important role in converting organic matter to readily biodegradable organic matter for organisms’ consumption, in which high molecular weight compounds such as protein, carbohydrates, and lipids are converted to a soluble phase. However, this stage is often restricted because of poor biodegradability of organic matter especially in anaerobic digestion of waste activated sludge [7]. To address this issue, recent researches have focused on different methods of enhancing the hydrolysis rate of anaerobic digestion of waste activated sludge with the thermal, chemical, and mechanical treatment of the sludge prior to anaerobic digestion [8–12]. These pretreatments disrupt sludge flocs, organisms’ cell walls, extracellular polymeric substance (EPS), intracellular organic matter, and multivalent cations, which increase biodegradability and hydrolysis rate of organic matter [13, 14]. According to recent studies, thermal pretreatment at a low temperature, alkaline pretreatment, and hydrogen peroxide pretreatment of sewage sludge have been considered as high-performance pretreatments in enhancing anaerobic digestion [10, 15].

Alkaline pretreatment is an effective method known in pretreatment methods, which can lead to the solubility of lignin and the types of neutralized acids produced by lignocellulosic complexes. In addition, the presence of a small amount of remaining alkali after pretreatment may help to stabilize pH during the acidogenesis process [16]. In many cases, as a post-digestion measure, lime as alkaline agent is added to the sludge so as to increase pH and kill pathogens, which improves the applicability of the sludge for different purposes such as fertilization [16].

Heat pretreatment especially at temperature above 100 °C is initially used to improve sludge dewaterability by destroying the gel structure, which can destroy cell walls to release organic chemicals into the soluble phase, resulting in higher biodegradation and lower sludge viscosity [17]. Thermal pretreatment is divided into two cases: low temperature (lower than 100 °C) and high temperature (higher than 100 °C). The pretreatment method, in which a lower temperature is used, is considered more economically attractive and environmentally friendly.

Hydrogen peroxide (H_2_O_2_) has been successfully used for disintegration of anaerobic biomass [18]; this is due to the production of free radicals such as NO•_2_ and HO• that disrupt cell walls, proteins, membrane phospholipids and EPS [19–21]. Interestingly, it has been revealed that H_2_O_2_ could be produced in situ from wastewater through a bio-electrochemical system [22]. Furthermore, Zhang et al. [23] reveal that H_2_O_2_ as a potential substance for combined pretreatments can increase methane production from waste activated sludge by 23%. Moreover, H_2_O_2_ causes the organic matter to enter the soluble phase and the microbial quality of sludge is slightly increased due to the lack of organic matter [14].

Therefore, thermal pretreatment was carried out in a warm water bath which was set at two specific temperatures. For alkaline pretreatment, by adding an adequate amount of the 1 N NaOH solution to the activated sludge which was filled in 1-l glasses, the pH of the containers was set at the particular numbers. In pretreatment with H_2_O_2_, values of 30 and 60 mg of H_2_O_2_/g TS were added to the activated sludge in the glasses. Moreover, in dual and triple pretreatments, the pretreatments were performed in a specific order in following with which was done for the single pretreatment.

This is the first study to analyze and optimize combined thermal, alkaline, and hydrogen peroxide pretreatment of waste activated sludge for enhancing anaerobic digestion of sewage sludge. In addition, the influence of various variables on methane production was analyzed using the response surface methodology (RSM) with the quadratic model. Eventually, an optimized temperature and chemical concentration for the highest methane production and lowest chemical usage is suggested.

## Results

### Source and characterization of sludge

Primary sludge, activated sludge, and inoculum were collected from South Tehran’s wastewater treatment plant. This plant is the largest wastewater treatment plant in the Middle East with the capacity of 450,000 m^3^/day to receive and process a sewage flow. Primary sludge from gravitational sedimentation, activated sludge from a belt thickener, and inoculum from mesophilic anaerobic digesters were collected for subsequent use. Primary and waste activated sludge were maintained at 4 °C and inoculum at 37 °C before being used in the experiments. Table 1 lists the main characteristics of primary sludge, waste activated sludge, and inoculum.

**Table 1:** Characteristics of sludge used in the experiment

Parameters	PS	WAS	Inoculum
TCOD (gL^− 1^)	35.94 ± 1.6	63.51 ± 3.5	37.00 ± 0.8
sCOD (gL^− 1^)	5.25 ± 0.2	4.81 ± 0.1	4.23 ± 0.1
TS (gL^− 1^)	27.91 ± 1.4	44.20 ± 1.6	29.06 ± 1.4
VS (gL^− 1^)	20.12 ± 0.4	35.41 ± 1.4	17.40 ± 0.3
TSS (gL^−1^)	23.82 ± 1.1	39.30 ± 1.9	22.14 ± 0.9
VSS (gL^−1^)	17.76 ± 0.9	30.11 ± 1.3	14.58 ± 0.2
pH	6.36 ± 0.1	6.52 ± 0.3	7.33 ± 0.2

*TCOD* total chemical oxygen demand, *sCOD* soluble chemical oxygen demand, *TS* total solid, *VS* volatile solids, *TSS* total suspended solids, *VSS* volatile suspended solids

### Pretreatment effect on organic matter solubilization

Considering the results of previous studies [4, 24, 25] and the effectiveness of the pre-tests performed in this study, the proposed input range, as shown in Table 2, was applied. For statistical analysis, variable levels were normalized to three levels low (0), medium (1), and high (2).

**Table 2:** Levels and code of variables

Variables	Symbols	Range and value of variables		
		0 (Low)	1 (Medium)	2 (High)
Temperature (°***C***)	A	25	75	90
Alkalinity (pH)	B	8	10	12
H_2_O_2_ concentration (mg/ g TS)	C	0	30	60

Table 3 shows the effects of different pretreatments on the solubility of organic matter in waste activated sludge. A significant increase in COD solubility in all treated samples was observed compared to the controls, varying from 3.65 to 30.37%. The highest increase in sCOD was due to the triple combination of A2 + B2 + C1 (19th test) pretreatment, which increased the sCOD to 19.29 g/L. Similar results are recorded in previous research [24, 26]. On the other hand, VSS variations are closely correlated with the sCOD. The highest VSS reduction, which was equal to 19.71 g/L, was also obtained from the combined pretreatment of A2 + B2 + C1. According to the ANOVA table for COD solubility (Additional file 1: Table S1), a second-order model with respect to R^2^ > 0.98 and *p*-value = 0.0003, which is smaller than the acceptable value of 0.05 has been suggested. According to the analysis and by eliminating the unwanted terms, the following equation is proposed for COD solubility:

1 $$ \mathrm{C}\mathrm{OD}\ \mathrm{solubilization}\left(\%\right)=\hbox{-} 67.011\hbox{-} 0.166\times \mathrm{A}+13.481\times \mathrm{B}+0.210\times \mathrm{C}\hbox{-} 0.017\times \mathrm{A}\times \mathrm{B}+0.004\times {\mathrm{A}}^2\hbox{-} 0.509\times {\mathrm{B}}^2\hbox{-} 0.003\times {\mathrm{C}}^2 $$

**Table 3:** Experimental design with response surface methodology and the results

No.	Input Variables			Results						
				Methane Production		Microbial				
	Temp. (°*C*)	pH	H_2_O_2_ concentration (mg/ g TS)	Cumulative MP (mL/ g VS)	Increase MP (%)	COD Solubilization (%)	Increase of sCOD (g/L)	Decrease of VSS (g/L)	Increase of Protein (g/L)	Increase of Polysaccharide (g/L)
1	25	8	0	314	0	3.65	2.32	1.95	0.52	0.01
2	25	8	30	373	18.79	8.09	5.14	5.11	0.95	0.03
3	25	8	60	358	14.01	9.05	5.75	4.25	1.35	0.04
4	25	10	30	410	30.57	18.67	11.86	10.61	2.16	0.27
5	25	10	60	426	35.67	18.97	12.05	12.24	2.53	0.22
6	25	12	0	444	41.4	14.56	9.25	8.67	1.66	0.17
7	25	12	60	471	50	22.59	14.35	13.88	2.94	0.18
8	75	8	30	402	28.02	16.72	10.62	10.98	1.68	0.22
9	75	8	30	415	32.17	16.34	10.38	11.21	1.84	0.2
10	75	10	30	480	52.67	21.67	13.76	14.35	2.87	0.38
11	75	10	30	469	49.36	21.82	13.86	14.51	2.46	0.34
12	75	10	30	488	55.41	21.56	13.69	14.2	2.81	0.28
13	75	10	60	506	61.15	23.43	14.88	15.72	2.97	0.33
14	75	10	60	502	59.87	23.51	14.93	15.99	3.02	0.23
15	75	12	0	492	56.69	17.86	11.34	12.01	2.39	0.17
16	90	8	0	418	33.12	15.24	9.68	8.08	2.14	0.18
17	90	8	60	524	66.88	22.17	14.08	15.64	2.85	0.31
18	90	10	0	479	52.55	21.1	13.4	14.55	2.74	0.36
19	90	12	30	615	95.86	30.37	19.29	19.47	3.81	0.52
20	90	12	30	621	97.77	30.17	19.16	19.71	3.91	0.59

According to the ANOVA table for VSS (Additional file 1: Table S1), each of the input variables had a significant effect on VSS solubility with R^2^ > 0.98 and *p*-value< 0.05. Interaction effects of B × C and C × A were removed because of their inappropriate *p*-value (> 0.05). According to the statistical analyses performed by the RSM and by eliminating the unwanted terms, the following equation is proposed for the VSS solubility:

2 $$ \mathrm{VSS}\ \mathrm{Solubilization}\left(\%\right)=-130.034-0.180\times \mathrm{A}+25.442\times \mathrm{B}+0.343\times \mathrm{C}-0.023\times \mathrm{A}\times \mathrm{B}+0.006\times {\mathrm{A}}^2-1.001\times {\mathrm{B}}^2-0.005\times {\mathrm{C}}^2 $$

In this study, the amount of soluble protein and soluble polysaccharide were measured before and after the treatments. According to the data obtained, the amount of soluble protein and soluble polysaccharide increased significantly when the pretreatments were applied to waste activated sludge. This enhancement was more considerable when a higher concentration of chemicals and higher temperature were used. The highest enhancement in soluble protein and soluble polysaccharide obtained from the bioreactor pre-treated with 20th test, in which they respectively reached to 3.91 g/L and 0.59 g/L. These values are considerably higher than those obtained from the control bioreactor with 0.52 g/L (soluble protein) and 0.01 g/L (soluble polysaccharide). The amount of soluble protein and soluble polysaccharide experienced a slight increase in the control bioreactor without any chemical addition or pH control, confirming a slight solubilization in the control due to natural activity of the organisms. A similar enhancement was observed in previous studies [12, 27]. For protein changes, the quadratic model is proposed with R^2^ > 0.97 and *p*-value = 0.0008 (Additional file 1: Table S1). The proposed model with the elimination of unwanted terminology is equal to:

3 $$ \mathrm{Increase}\ \mathrm{of}\ \mathrm{Protein}\ \left(\mathrm{g}/\mathrm{L}\right)=\hbox{-} 8.071\hbox{-} 0.042\times \mathrm{A}+1.728\times \mathrm{B}+0.012\times \mathrm{C}+0.002\times \mathrm{B}\times \mathrm{C}+0.001\times {\mathrm{A}}^2\hbox{-} 0.070\times {\mathrm{B}}^2\hbox{-} 0.0002\times {\mathrm{C}}^2 $$

**Fig. 1:**
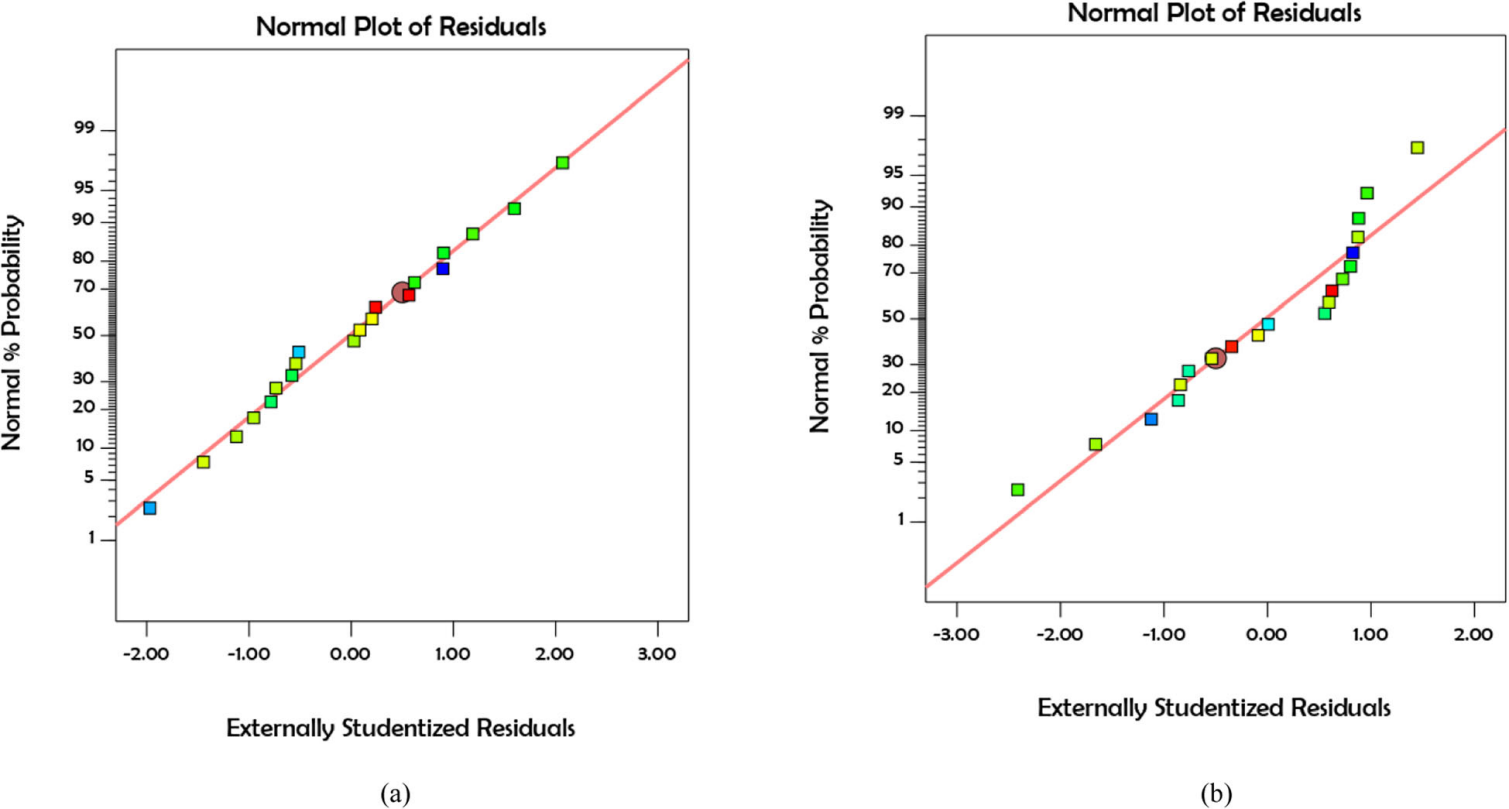
Normal plots of residuals for **a** COD solubilization **b** increase of protein

**Fig. 2:**
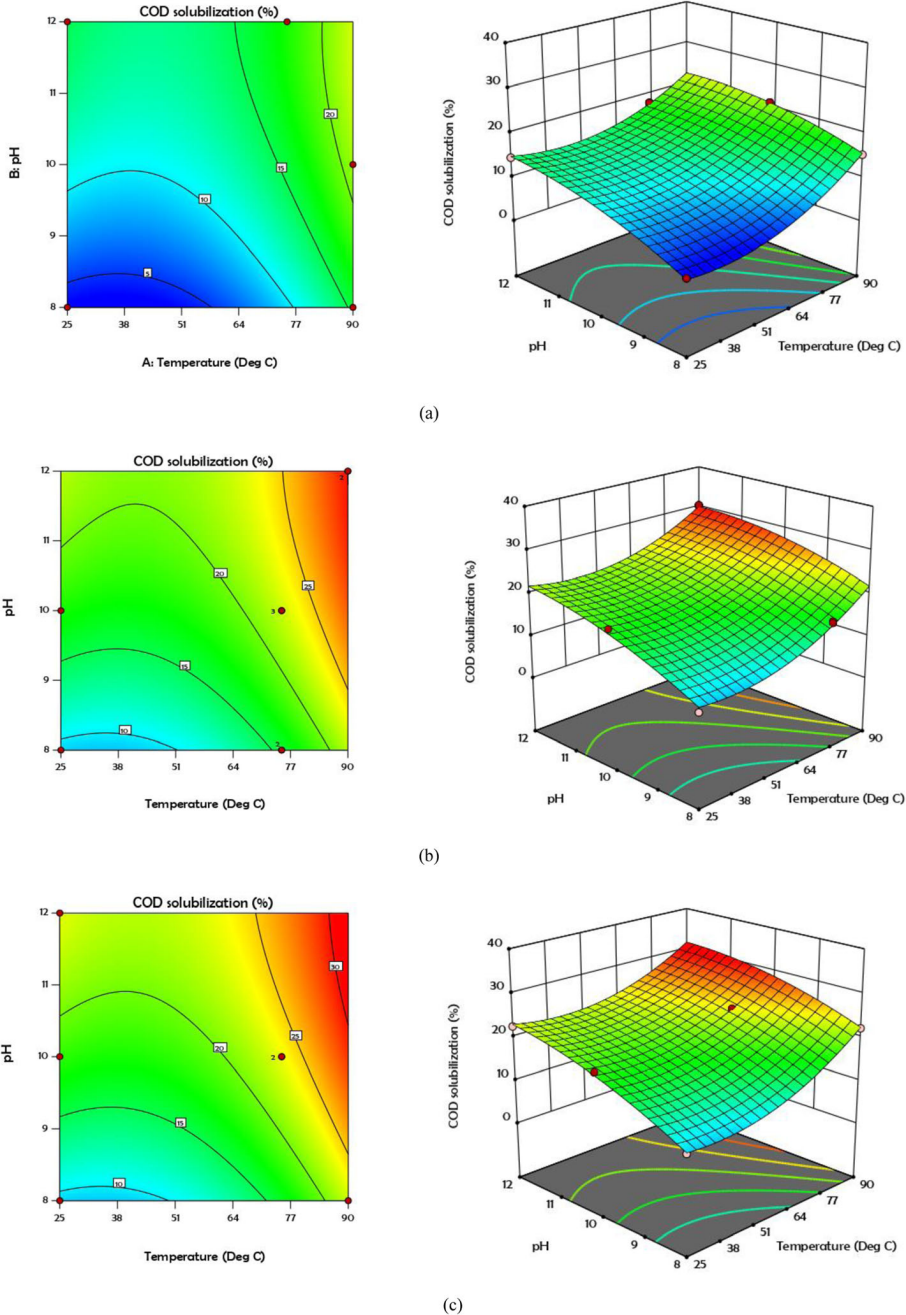
Contour and 3D plots for COD solubilization at different concentration of H_2_O_2_**a** 0 **b** 30 and **c** 60 mg/g TS

**Fig. 3:**
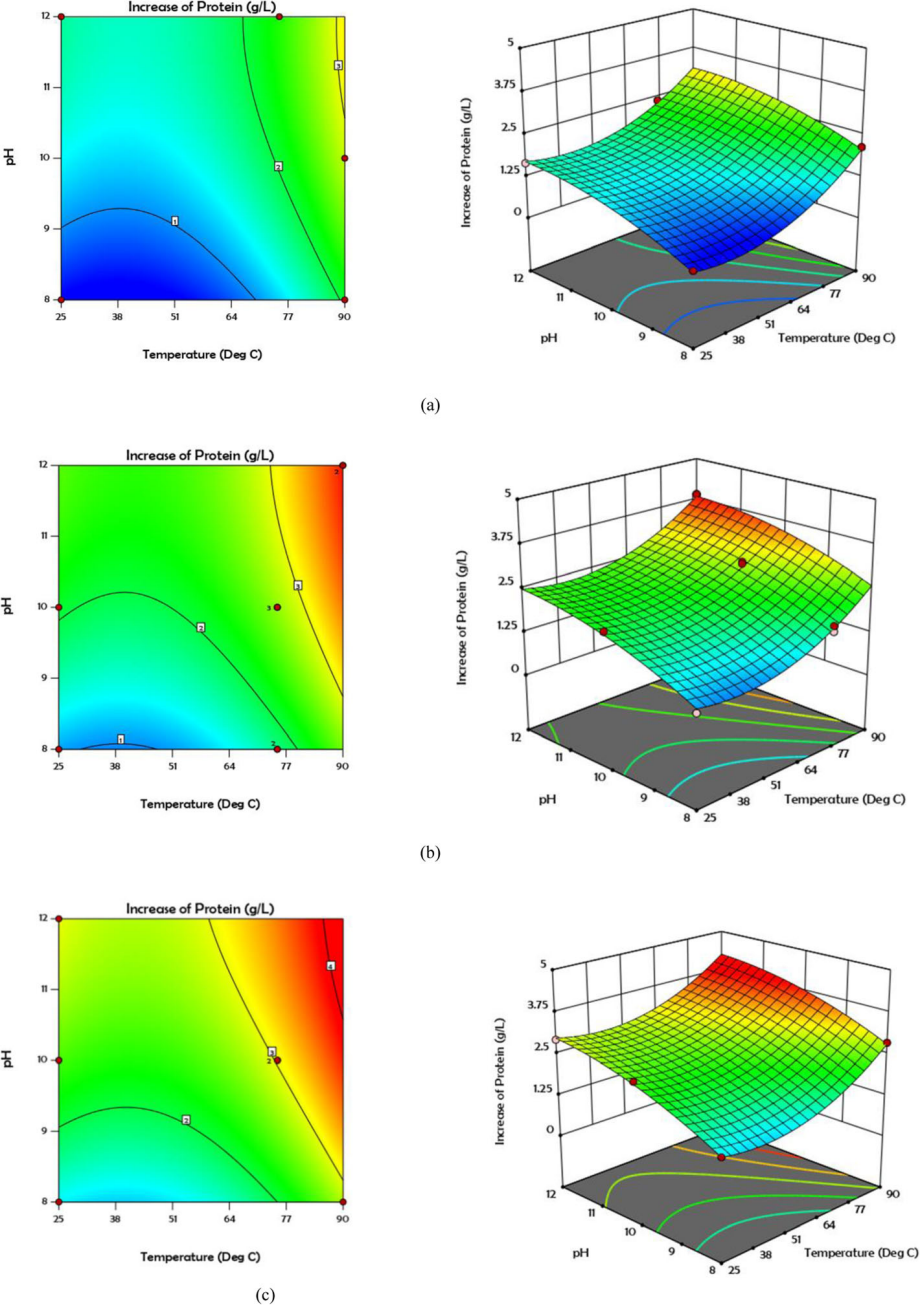
Contour and 3D plots for Increase of protein at different concentration of H_2_O_2_**a** 0 **b** 30 and **c** 60 mg/g TS

Figure 1 shows the normal probability figures of COD and protein solubility. According to these forms, the less the dispersion of the existing data, the closer the results are to the normal line. In other words, when the *R*^2^ value tends to 1, the proposed model is stronger. Contour and 3D curves for COD and protein solubility at various concentrations of 0, 30, and 60 mg H_2_O_2_/g TS are shown in Figs. 2 and 3. According to Fig. 2, COD solubility is predicted to increase up to 32% in combined pretreatment of 60 mg H_2_O_2_/g TS, 90 °C, and pH = 12, while the highest solubility enhancement from experimental results was 30.4% (19th test). The highest predicted VSS solubility was 55% in combined pretreatment of 60 mg H_2_O_2_/g TS, pH = 12, and 90 °C, while the highest amount obtained from experimental results was 50% obtained from the 20th test (30 mg H_2_O_2_/g TS, pH = 12, and 90 °C). The addition of hydrogen peroxide to pretreatment demonstrated its function in breaking down the cell walls, as well as the EPS, caused by the release of radicals (hydroxyl radicals, hydroperoxyl radicals), converting organic matter to soluble phase [14].

### Daily biogas production

**Fig. 4:**
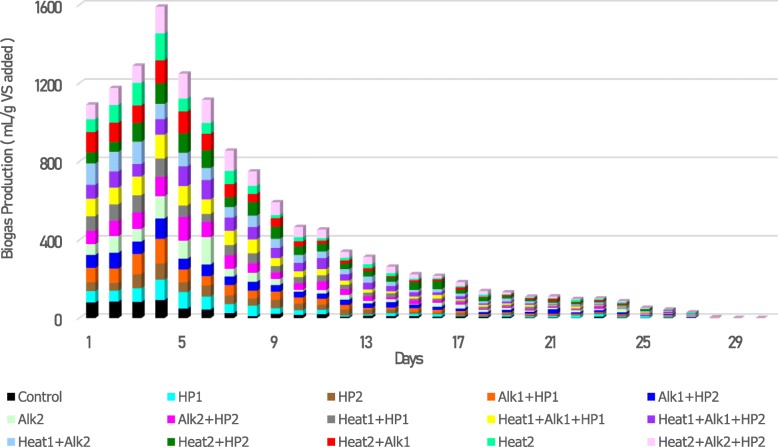
Daily specific biogas production

**Fig. 5:**
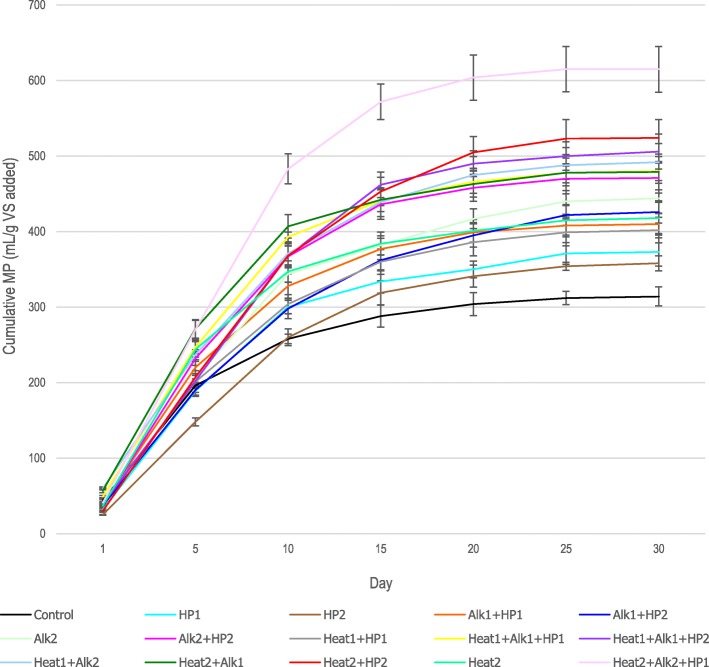
Effect of pretreatments on cumulative methane production. Error bars represent standard errors gained from triplicate measurements

As shown in Figs. 4 and 5, the highest daily biogas production was obtained between third and fifth days through the bioreactors. During the first days of the digestion process (Additional file 1: Table S2), the amount of biogas production in the control bioreactor was higher than most of the pre-treated bioreactors. This can be either attributed to the presence of inhibitory factors of the pretreatment or due to the high organic loading available to anaerobic organisms and excessive volatile fatty acid [28].

### Cumulative methane production

The amount of methane production from different bioreactors was measured regularly during the anaerobic digestion process (Additional file 1: Table S3). For normalizing the data, the produced methane (mL) was divided by the added volatile solids (gram) to each bioreactor. The effects of different pretreatment types on the cumulative yield of biogas production are shown in Table 3. The amount of cumulative methane yield was considerably enhanced when combined pretreatment methods were used. The highest enhancement in methane production (97.77%), compared to the control, was obtained when the combination of A2 + B2 + C1 pretreatments was employed. This is 30.89% higher than the highest increase achieved from the bioreactors with dual treatments (A2 + C2), corroborating the effectiveness of triple pretreatment compared to individual and dual pretreatments. As in previous studies [13, 25, 29], the methane enhancement considering only the individual pretreatment used in this study was between 10 and 30%. In this study, the methane increase from individual pretreatment was between 14% in C2 and 33% in A2. Methane enhancement from dual pretreatments was between 28 and 66%, while in previous studies, it was around 20 to 70% [12, 24].

Analysing the results of cumulative biogas production in Table 3 using RSM, a quadratic model with R^2^ > 0.98 and *p*-value< 0.0001 was suggested (Additional file 1: Table S1). By eliminating unacceptable terms and incorporating acceptable terms, the following equation was achieved:

4 $$ \mathrm{Increase}\ \mathrm{MP}\ \left(\%\right)=\hbox{-} 39.897\hbox{-} 1.798\times \mathrm{A}+8.408\times \mathrm{B}+0.678\times \mathrm{C}+0.005\times \mathrm{A}\times \mathrm{C}+0.0181\times {\mathrm{A}}^2\hbox{-} 0.006\times {\mathrm{C}}^2 $$

**Fig. 6:**
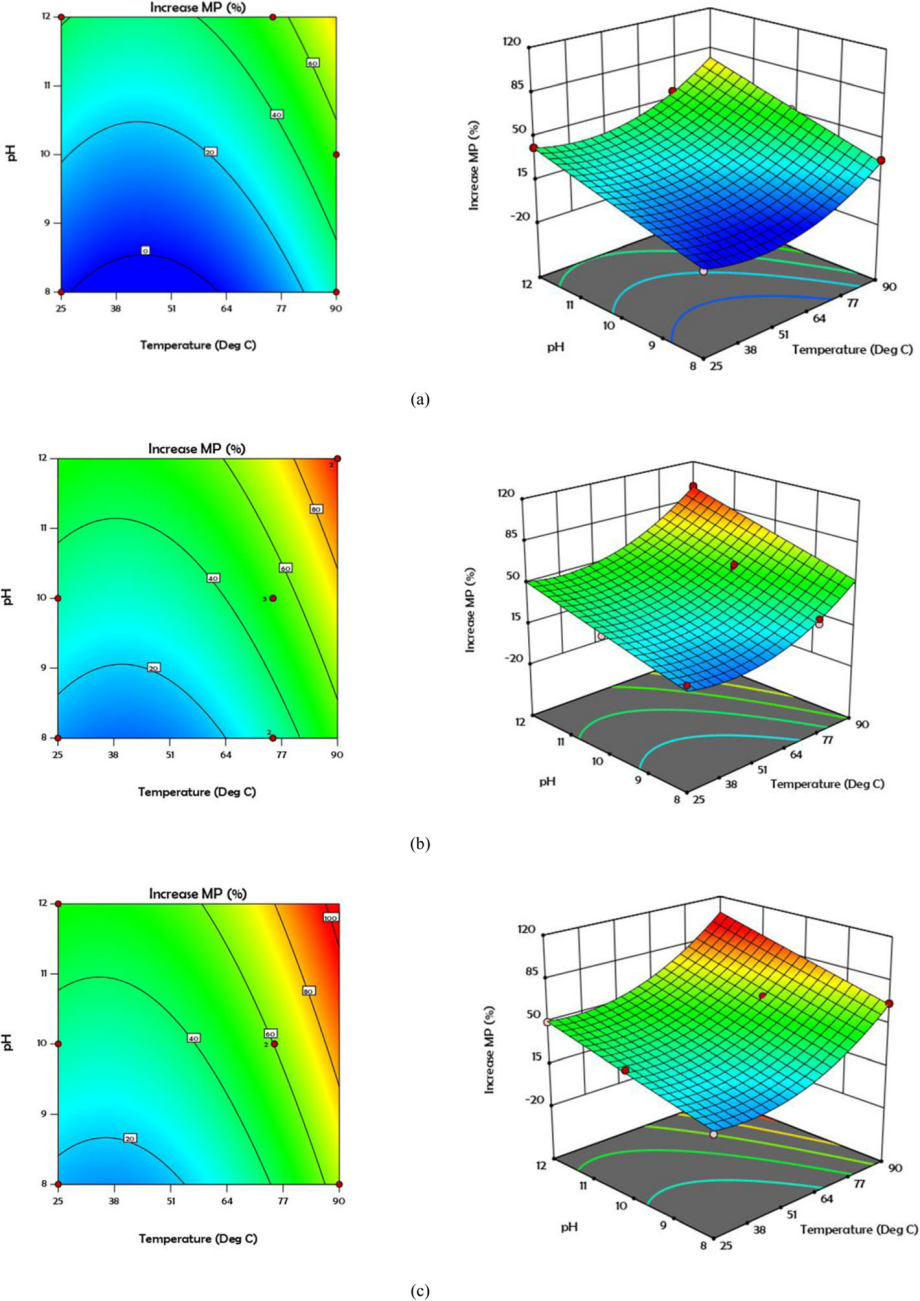
Contour and 3D plots for MP at different concentration of H_2_O_2_**a** 0 **b** 30 and **c** 60 mg/g TS

Figure 6 shows contour and 3D graphs related to the increase of cumulative methane production in different concentrations of hydrogen peroxide. According to these diagrams, the highest percentage of cumulative methane production is about 115%, which abstained from the bioreactors pre-treated with 90 °C, pH = 12, and 60 mg H_2_O_2_/g TS. It is important to note that the highest increase in cumulative biogas production, which was 98%, was observed in the 20th test.

### Optimum pretreatment condition

A scenario was written for achieving the optimum condition, whereby temperature, pH, and H_2_O_2_ were minimized and the methane production, sCOD, soluble protein, and soluble polysaccharide were maximized. In the scenario, methane production was allocated the highest importance factor (Table 4). The best-suggested desirability was 0.673. Desirability is an objective function that varies from zero outside the range to one in the goal. Numerical optimization finds a point that maximizes utility performance. The characteristics of a goal may be changed by adjusting the weight or importance. For several responses and factors, all goals fall into one desirable function [30]. Table 4 shows the information pertaining to the optimization process. Another test with the suggested inputs was carried out for verifying the predicted phenomena in the model, in which the methane enhancement of 71% was achieved in the pre-treated bioreactor (Table 5). The difference achieved can be attributed to different sludge characterizations in the verification test. Despite the important results this study represents, applying new systems to anaerobic digestion of sewage sludge entails precise economic and feasibility assessments. Thus, in prospective studies, economic assessments for full-scale application of the pretreatments are crucial, in addition to investigating possible side-effects of the pretreatments on microbial communities and behavior in long-term exposure.

**Table 4:** Constraints of optimum condition

Name	Goal	Lower Limit	Upper Limit	Lower Weight	Upper Weight	Importance
A: Temperature	Minimize	75	90	1	1	4
B: pH	Minimize	8	12	1	1	4
C: H_2_O_2_ Concentration	Minimize	0	60	1	1	4
Cumulative MP	Maximize	314	621	1	1	5
Increase MP	Maximize	0	97.77	1	1	5
Increase of sCOD	Maximize	2.32	19.29	1	1	4
COD solubilization	Maximize	3.65	30.37	1	1	4
Decrease of VSS	Maximize	1.95	19.71	1	1	3
VSS solubilization	Maximize	4.96	50.15	1	1	3
Increase of Protein	Maximize	0.52	3.91	1	1	5
Increase of Polysaccharide	Maximize	0.01	0.59	1	1	2

**Table 5:** Predicted and actual observation data at the achieved optimum pretreatment

Input Variables			Result															
Temp (°*C*)	pH	H_2_O_2_ Concentr-ation (mg/g TS)	Cumulative MP (mL/g VS)		Increase MP (%)		Increase of sCOD (g/g VS)		COD solubiliza-tion (%)		Decrease of VSS (g/g VS)		VSS solubilization (%)		Increase of protein (g/L)		Increase of polysaccharide (g/L)	
83.2	10.6	34.8	Pre	Act	Pre	Act	Pre	Act	Pre	Act	Pre	Act	Pre	Act	Pre	Act	Pre	Act
			542	529	72.6	71.1	17.1	17.9	26.9	28.2	17.9	18.1	45.4	45.7	3.35	3.55	0.46	0.42

## Discussion

The main objective of this research was to investigate the effect of combined and individual thermal, alkaline, and hydrogen peroxide pretreatment of waste activated sludge on anaerobic digestion efficiency.

The significant increase of COD solubility can be attributed to the breakdown of cellular and microbial walls and the release of organic materials such as polysaccharides and proteins to the soluble phase [31, 32]. All three input variables of temperature, pH, and H_2_O_2_ concentration individually affected the COD solubility significantly, whose *p*-values were less than < 0.0001. For determining the effects of interactions, the term A × B had a positive effect with *p*-value< 0.05 and appropriate F-value, but the effect of C × B and C × A was insignificant with *p*-value> 0.1 and the very low and inappropriate F-value, so they were deleted. For the second-order equation, A^2^, B^2^, and C^2^ had significant effects (*p*-value< 0.05). Also, according to ANOVA table (Additional file 1: Table S1), the model is very suitable because of the lack of fit *p*-value and fit F-value. Organic matter is generally divided into two parts: biodegradable organic matter, which is consumed by microbial community, and nonbiodegradable organic matter, which is not decomposed by organisms. Protein and polysaccharide, the two readily biodegradable components of COD, account for around 60% of organisms constituents in sludge [3]. Therefore, enhancement of soluble protein and polysaccharide means that a higher amount of nutrients is available to the anaerobic organisms. The increase in amount of soluble protein was considerably higher than that of soluble polysaccharide after treatments can be attributed to a higher proportion of protein in organisms constituents (around 50%) [3]. Thus, the pretreatments probably disrupted cell walls and EPS in waste activated sludge due to their biocidal effect, which affects protein much more than polysaccharide. This agrees with previous studies, in which a similar trend is observed [12, 33]. All three input variables, with *p*-value< 0.05, had a significant effect on the model, while the effect of the input parameters on each other will be ignored due to *p*-value> 0.05. Meanwhile, second-order semigroups of input parameters, due to *p*-value < 0.05 and high F-value, could have a positive effect on protein changes (Additional file 1: Table S1).

The enhanced methane is of paramount importance because not only does it enhance renewable energy generation in wastewater treatment plants, but it also reduces methane emission to the atmosphere, as a major greenhouse gas emission [34]. According to ANOVA table (Additional file 1: Table S1), all three input variables significantly affect methane production, yet their interactions did not affect the methane production substantially. The second-order effects of the terms A^2^ and C^2^ had a particular effect on the increase of methane production with *p*-value< 0.05, while B^2^ was eliminated from the model with *p*-value> 0.1.

## Conclusion

This study investigated the possibility of enhancing anaerobic digestion of sewage sludge with different pretreatments. It was revealed that the triple pretreatment of waste activated sludge with heat, alkalinity, and hydrogen peroxide increases soluble fractions of organic matter considerably more than dual and individual pretreatments. This led to significantly higher daily biogas and methane production from the anaerobic digestion, as a higher amount of biodegradable organic matter was available to the anaerobic microbial community. In addition, the effect of input variables and their interactions on methane production were analyzed with response surface methodology and optimized input variables were suggested in the end. Furthermore, harnessing a higher amount of methane in the anaerobic digestion stage decreases methane emission to the atmosphere in dewatering and landfilling stages and enhance the quality of digested sludge, bringing about environmentally friendly and economically attractive sewage sludge treatment process.

## Methods

### Statisical analyses and DOE

RSM was used to evaluate the independent, interactive, and quadratic input parameters (temperature, pH, and hydrogen peroxide concentration). The I-optimal method was also used for finding optimum points for the highest methane production and organic matter solubilization. In mixture experiments, the proportions of the components of a mixture are the studied factors. The special nature of the factors makes special kinds of regression models necessary and special kinds of experimental designs. Although mixture experiments usually are to foresee the response(s) for all potential formulations of the mixture and to recognize optimal amounts for each of the ingredients, little research has been done regarding their I-optimal design. That I-optimal designs decreases the average variance of prediction and, consequently, looks more suitable for mixture experiments than the commonly used D-optimal designs, focusing on an exact model estimation rather than exact predictions. Also, the I-optimal method gives the ability to consider all relevant points in the desired range regardless of the intermediate data, with three specific points [30].

Design-Expert® software (version 11.0.5.0) was used for statistical analysis and to find the optimal answer. The three considered factors and their interactions were analyzed using the ANOVA table. To determine the significance of differences in the parameters studied, one-way factor Analysis of Variance ANOVA was used with significance levels of *p* < 0.05. Data analysis and graph processing were carried out with Microsoft Excel software (2010).

### Pretreatment methods

The different types of pretreatment methods such as thermal, alkaline and hydrogen peroxide pretreatments were applied individually and in combination with the experimental design shown in Table 3, in accordance with the previous studies as well as their performance. Thermal pretreatment was performed for 5 h in a warm water bath that was set at two temperatures of 75 °C and 90 °C. For this purpose, 1-l sealed glasses containing activated sludge were used. For alkaline pretreatment, since the pH of the primary activated sludge is about 7, 1 N NaOH solution, which represents a high impact alkalinity, as well as a reasonable price, was used to increase the pH of the activated sludge. Thus, by adding a sufficient amount of the solution to the activated sludge which was filled in 1-l glasses, the pH of the containers was adjusted to 8, 10 and 12. After adjusting the desired pH, each container was placed on the shaker at 150 rpm for a 24 h residence time to keep the sludge uniform over the retention time. In pretreatment with hydrogen peroxide 30% (w/w) merck, values of 30 and 60 mg of H_2_O_2_/g TS were added to the activated sludge in the 1-l glasses. Then, the glasses, which contain the specific amounts of hydrogen peroxide, were placed on a shaker at the 150 rpm for 24 h to keep the sludge uniform over the retention time. Also, in combined pretreatments, the pretreatments were performed in a specific order in accordance with what was done for the single pretreatment.

### Anaerobic digestion

The designed biochemical methane potential test consisted of a 1-l glass reactor containing 600 ml of mixed primary and pre-treated waste activated sludge with a 70%:30% (w/w). The same ratio between primary and waste activated sludge is considered in the South Tehran wastewater treatment plant. Liquid displacement method was used for measuring the volume of biogas production [35]. Strict anaerobic conditions were provided for better organism performance. The reactors were kept in a mesophilic environment at 37 °C, using a hot water bath heated by automatic heaters. During anaerobic digestion, the mixture was stirred at 100 rpm using magnetic stirrers for retaining uniform temperature and nutrient distribution. All measurements were carried out in triplicate with standard error as dispersion measure. Anaerobic digestion process continued for 30 days when biogas production was negligible.

### Analytical method

Chemical oxygen demand (COD), total solid (TS), total suspended solid (TSS), volatile solid (VS), and volatile suspended solid (VSS) were measured according to standard methods for examinations of water and wastewater [36]. The COD and VSS solubility after pretreatment were obtained through the following equation:

5 $$ \mathrm{COD}\ \mathrm{solubilization}=\frac{{\mathrm{sCOD}}_{\mathrm{t}}-{\mathrm{sCOD}}_0}{{\mathrm{TCOD}}_0}\times 100 $$

6 $$ \mathrm{VSS}\ \mathrm{solubilization}=\frac{{\mathrm{VSS}}_0-{\mathrm{VSS}}_{\mathrm{t}}}{{\mathrm{TSS}}_0}\times 100 $$

Where *t* represents the value after pretreatment and 0 indicates the initial state.

To separate soluble solids from suspensions, centrifuges were used at a speed of 10,000 rpm for 30 min, and filters with a porosity of 0.45 μm were used. Soluble proteins were analyzed with Folin Phenol in accordance with Lowry’s study [37], and soluble polysaccharides were analyzed with phenol and sulfuric acid based on the Dubois study [38]. The percentage of methane was measured using gas chromatography (GC) with a thermal conductivity detector (TCD) at 100 °C and an oven at 60 °C. For the tests, 1 mL of the samples were injected into the GC device.

## Supplementary information

**Additional file 1: Table S1.** ANOVA for Quadratic model of Increase MP, COD and VSS solubilization and Protein. **Table S2.** Daily biogas production (mL/g VS added) (average of triplicate tests). **Table S3.** Cumulative methane production (mL/g VS added).

## Data Availability

All data generated or analyzed during this study are included in this published article and its supplementary information files.

## Abbreviations

EPS: Extracellular polymeric substance
RSM: Response surface methodology
PS: Primary sludge
WAS: Waste active sludge
TCOD: Total chemical oxygen demand
sCOD: Soluble chemical oxygen demand
TS: Total solid
VS: Volatile solid
TSS: Total suspended solid
VSS: Volatile suspended solid
MP: Methane production
GC: Gas chromatography
TCD: Thermal conductivity detector
