# Supplementary material for: Process optimization and effect of thermal, alkaline, H2O2 oxidation and combination pretreatment of sewage sludge on solubilization and anaerobic digestion
Source: BMC Biotechnol. 2020 May 6;20:21. doi: 10.1186/s12896-020-00614-1 (PMC7201573; doi:10.1186/s12896-020-00614-1)
Supplement: Supplementary file 1 — Additional file 1: Table S1. ANOVA for Quadratic model of Increase MP, COD and VSS solubilization and Protein. Table S2. Daily biogas production (mL/g VS added) (average of triplicate tests). Table S3. Cumulative methane production (mL/g VS added). [file 12896_2020_614_MOESM1_ESM.docx]

Table S1. ANOVA for Quadratic model of Increase MP, COD and VSS solubilization and Protein

| Source | Increase MP | | | COD Solubilization | | | VSS solubilization | | | Increase Protein | | |
| --- | --- | --- | --- | --- | --- | --- | --- | --- | --- | --- | --- | --- |
|  | F-value | p-value |  | F-value | p-value |  | F-value | p-value |  | F-value | p-value |  |
| Model | 156.01 | < 0.0001 |  | 139.43 | < 0.0001 |  | 55.47 | < 0.0001 |  | 83.95 | < 0.0001 |  |
| A- Temperature | 631.40 | < 0.0001 |  | 444.77 | < 0.0001 |  | 195.48 | < 0.0001 |  | 303.51 | < 0.0001 |  |
| B- pH | 554.40 | < 0.0001 |  | 448.35 | < 0.0001 |  | 153.65 | < 0.0001 |  | 267.59 | < 0.0001 |  |
| C- H_2_O_2_ Concentration | 148.82 | < 0.0001 |  | 207.17 | < 0.0001 |  | 80.30 | < 0.0001 |  | 133.45 | < 0.0001 |  |
| AB | 2.45 | 0.1488 |  | 16.13 | 0.0025 |  | 3.78 | 0.0805 |  | 1.05 | 0.3289 |  |
| AC | 23.83 | 0.0006 |  | 0.6213 | 0.4489 |  | 2.62 | 0.1366 |  | 0.5105 | 0.4913 |  |
| BC | 1.57 | 0.2387 |  | 2.97 | 0.1158 |  | 0.2857 | 0.6046 |  | 3.41 | 0.0948 |  |
| A² | 69.18 | < 0.0001 |  | 46.15 | < 0.0001 |  | 10.87 | 0.0081 |  | 35.74 | 0.0001 |  |
| B² | 0.0176 | 0.8971 |  | 23.13 | 0.0007 |  | 11.09 | 0.0076 |  | 15.75 | 0.0027 |  |
| C² | 14.51 | 0.0034 |  | 49.80 | < 0.0001 |  | 13.31 | 0.0045 |  | 5.35 | 0.0433 |  |
| Lack of Fit | 1.69 | 0.2895 |  | 50.53 | 0.0003 |  | 48.76 | 0.0003 |  | 0.5874 | 0.7132 |  |

Table S2. Daily biogas production (mL/g VS added) (average of triplicate tests)

|  | Control | HP1 | HP2 | Alk1  HP1 | Alk1  HP2 | Alk2 | Alk2  HP2 | Heat1  HP1 | Heat1  Alk1  HP1 | Heat1  Alk1  HP2 | Heat1  Alk2 | Heat2  HP2 | Heat2  Alk1 | Heat2 | Heat2  Alk2  HP2 |
| --- | --- | --- | --- | --- | --- | --- | --- | --- | --- | --- | --- | --- | --- | --- | --- |
| 1 | 79.5 | 58.4 | 45.5 | 73.1 | 66.6 | 55.2 | 68.2 | 74.7 | 89.3 | 71.4 | 108.8 | 55.2 | 103.9 | 66.6 | 74.7 |
| 2 | 84.4 | 55.2 | 40.6 | 73.1 | 81.2 | 84.4 | 79.5 | 82.8 | 86 | 82.8 | 99 | 50.3 | 99 | 90.9 | 86 |
| 3 | 82.8 | 69.8 | 73.1 | 102.3 | 63.3 | 64.9 | 84.4 | 87.7 | 95.8 | 63.3 | 113.6 | 95.8 | 89.3 | 115.3 | 87.7 |
| 4 | 92.5 | 103.9 | 84.4 | 125 | 103.9 | 113.6 | 99 | 92.5 | 121.8 | 79.5 | 77.9 | 103.9 | 118.5 | 138 | 136.4 |
| 5 | 48.7 | 84.4 | 50.3 | 64.9 | 55.2 | 92.5 | 121.8 | 58.4 | 99 | 100.6 | 68.2 | 99 | 112 | 66.6 | 126.6 |
| 6 | 43.8 | 66.6 | 56.8 | 47.1 | 60.1 | 139.6 | 77.9 | 40.6 | 74.7 | 99 | 61.7 | 89.3 | 84.4 | 55.2 | 118.5 |
| 7 | 26 | 45.5 | 45.5 | 51.9 | 43.8 | 39 | 69.8 | 51.9 | 73.1 | 68.2 | 53.6 | 50.3 | 66.6 | 68.2 | 102.3 |
| 8 | 9.7 | 53.6 | 37.3 | 39 | 45.5 | 47.1 | 48.7 | 50.3 | 71.4 | 63.3 | 58.4 | 68.2 | 42.2 | 42.2 | 73.1 |
| 9 | 21.1 | 29.2 | 43.8 | 40.6 | 34.1 | 30.8 | 34.1 | 32.5 | 40.6 | 51.9 | 47.1 | 61.7 | 43.8 | 16.2 | 64.9 |
| 10 | 16.2 | 24.4 | 32.5 | 32.5 | 29.2 | 9.7 | 34.1 | 30.8 | 29.2 | 43.8 | 40.6 | 43.8 | 26 | 21.1 | 51.9 |
| 11 | 19.5 | 22.7 | 26 | 30.8 | 26 | 17.9 | 43.8 | 32.5 | 30.8 | 56.8 | 34.1 | 39 | 16.2 | 13 | 43.8 |
| 12 | 6.5 | 6.5 | 30.8 | 22.7 | 27.6 | 24.4 | 29.2 | 22.7 | 21.1 | 32.5 | 26 | 29.2 | 16.2 | 13 | 30.8 |
| 13 | 11.4 | 6.5 | 24.4 | 9.7 | 22.7 | 13 | 24.4 | 17.9 | 16.2 | 34.1 | 32.5 | 27.6 | 14.6 | 21.1 | 37.3 |
| 14 | 9.7 | 14.6 | 19.5 | 9.7 | 27.6 | 14.6 | 11.4 | 19.5 | 9.7 | 27.6 | 21.1 | 22.7 | 6.5 | 16.2 | 32.5 |
| 15 | 9.7 | 11.4 | 11.4 | 17.9 | 17.9 | 3.2 | 19.5 | 9.7 | 9.7 | 21.1 | 13 | 37.3 | 11.4 | 8.1 | 22.7 |
| 16 | 6.5 | 13 | 8.1 | 14.6 | 16.2 | 19.5 | 13 | 9.7 | 17.9 | 13 | 16.2 | 40.6 | 9.7 | 6.5 | 11.4 |
| 17 | 8.1 | 3.2 | 14.6 | 9.7 | 13 | 19.5 | 11.4 | 9.7 | 6.5 | 16.2 | 13 | 19.5 | 13 | 6.5 | 19.5 |
| 18 | 6.5 | 3.2 | 3.2 | 6.5 | 11.4 | 11.4 | 4.9 | 11.4 | 8.1 | 8.1 | 11.4 | 17.9 | 4.9 | 11.4 | 17.9 |
| 19 | 6.5 | 8.1 | 8.1 | 6.5 | 13 | 9.7 | 6.5 | 8.1 | 8.1 | 9.7 | 16.2 | 11.4 | 6.5 | 3.2 | 9.7 |
| 20 | 3.2 | 4.9 | 8.1 | 6.5 | 9.7 | 6.5 | 6.5 | 13 | 8.1 | 8.1 | 11.4 | 9.7 | 6.5 | 3.2 | 3.2 |
| 21 | 3.2 | 6.5 | 4.9 | 6.5 | 24.4 | 6.5 | 4.9 | 6.5 | 3.2 | 4.9 | 6.5 | 9.7 | 11.4 | 3.2 | 8.1 |
| 22 | 1.6 | 11.4 | 3.2 | 4.9 | 8.1 | 11.4 | 4.9 | 4.9 | 9.7 | 3.2 | 6.5 | 8.1 | 4.9 | 11.4 | 3.2 |
| 23 | 6.5 | 13 | 9.7 | 1.6 | 9.7 | 6.5 | 11.4 | 4.9 | 3.2 | 4.9 | 8.1 | 6.5 | 6.5 | 4.9 | 3.2 |
| 24 | 3.2 | 3.2 | 9.7 | 3.2 | 9.7 | 17.9 | 0 | 6.5 | 3.2 | 3.2 | 3.2 | 4.9 | 6.5 | 6.5 | 4.9 |
| 25 | 1.6 | 8.1 | 0 | 1.6 | 3.2 | 3.2 | 3.2 | 3.2 | 4.9 | 3.2 | 3.2 | 8.1 | 1.6 | 3.2 | 3.2 |
| 26 | 3.2 | 3.2 | 4.9 | 4.9 | 4.9 | 3.2 | 0 | 0 | 0 | 8.1 | 4.9 | 0 | 3.2 | 3.2 | 0 |
| 27 | 0 | 0 | 3.2 | 0 | 3.2 | 4.9 | 0 | 4.9 | 1.6 | 3.2 | 3.2 | 1.6 | 0 | 3.2 | 0 |
| 28 | 0 | 0 | 0 | 0 | 0 | 0 | 1.6 | 0 | 1.6 | 0 | 0 | 0 | 0 | 0 | 0 |
| 29 | 0 | 0 | 0 | 0 | 0 | 0 | 0 | 0 | 0 | 0 | 0 | 0 | 0 | 0 | 0 |
| 30 | 0 | 0 | 0 | 0 | 0 | 0 | 0 | 0 | 0 | 0 | 0 | 0 | 0 | 0 | 0 |

Table S3. Cumulative methane production (mL/g VS added)

|  | **Control** |  |  |  |  |  |  | **Heat1** | **Heat1** | **Heat1** | **Heat1** | **Heat2** | **Heat2** | **Heat2** | **Heat2** |
| --- | --- | --- | --- | --- | --- | --- | --- | --- | --- | --- | --- | --- | --- | --- | --- |
|  |  |  |  | **Alk1** | **Alk1** | **Alk2** | **Alk2** |  | **Alk1** | **Alk1** | **Alk2** |  | **Alk1** |  | **Alk2** |
|  |  | **HP1** | **HP2** | **HP1** | **HP2** |  | **HP2** | **HP1** | **HP1** | **HP2** |  | **HP2** |  |  | **HP2** |
| **1** | 44 | 32 | 25 | 40 | 37 | 30 | 38 | 41 | 49 | 39 | 60 | 30 | 57 | 37 | 41 |
| **5** | 196 | 189 | 148 | 220 | 190 | 206 | 232 | 201 | 247 | 202 | 237 | 207 | 271 | 244 | 270 |
| **10** | 258 | 300 | 260 | 328 | 298 | 344 | 367 | 304 | 393 | 368 | 371 | 368 | 407 | 347 | 483 |
| **15** | 288 | 334 | 319 | 377 | 362 | 382 | 436 | 360 | 441 | 462 | 439 | 453 | 442 | 384 | 572 |
| **20** | 304 | 350 | 341 | 399 | 395 | 417 | 458 | 386 | 466 | 490 | 475 | 505 | 463 | 401 | 604 |
| **25** | 312 | 371 | 354 | 408 | 422 | 440 | 470 | 399 | 478 | 500 | 488 | 523 | 478 | 415 | 615 |
| **30** | 314 | 373 | 358 | 410 | 426 | 444 | 471 | 402 | 480 | 506 | 492 | 524 | 479 | 418 | 615 |
